# Supplementary material for: The genomic evolutionary dynamics and global circulation patterns of respiratory syncytial virus
Source: Nat Commun. 2024 Apr 10;15:3083. doi: 10.1038/s41467-024-47118-6 (PMC11006891; doi:10.1038/s41467-024-47118-6)
Supplement: Supplementary file 1 — Supplementary information [file 41467_2024_47118_MOESM1_ESM.docx]

**Supplementary Figures**

**Figure S1**: Phylogenetic summary of the amino acid substitutions at positively selected sites (as supported by at least two of these methods) for RSVA. The thickness of each branch in the Maximum Clade Credibility (MCC) tree corresponds to the number of AA changes that occurred over that branch as indicated in the legend. Branch colours identify the number of AA changes and affected protein(s), with correspondence as in the legend. The number of AA changes and affected protein(s) are also indicated next to the relevant lineage. Lineages that did not accommodate a nonsynonymous change are depicted in light grey.

**Figure S2**: Phylogenetic summary of the amino acid substitutions at positively selected sites (as supported by at least two of these methods) for RSVB. The thickness of each branch in the Maximum Clade Credibility (MCC) tree corresponds to the number of AA changes that occurred over that branch as indicated in the legend. Branch colours identify the number of AA changes and affected protein(s), with correspondence as in the legend. The number of AA changes and affected protein(s) are also indicated next to the relevant lineage. Lineages that did not accommodate a nonsynonymous change are depicted in light grey.

**
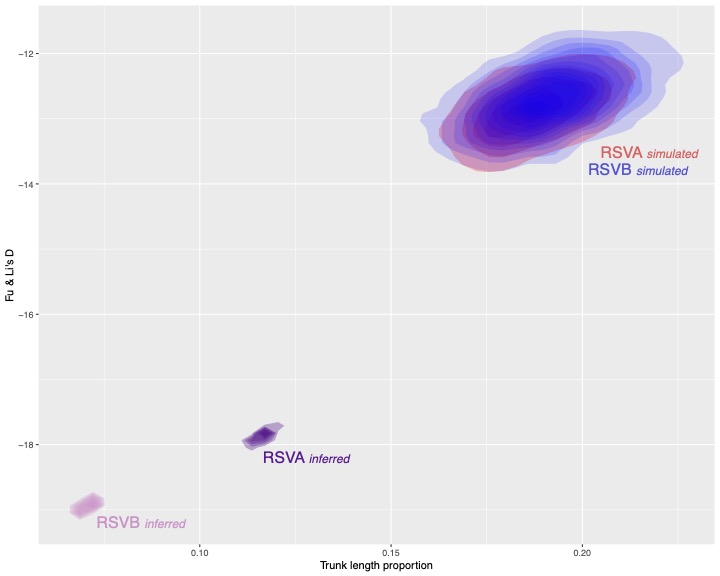
**

**Figure S3**: Comparison of tree shape statistics derived from inferred and simulated RSV A and B genealogies. Contours filled with colors of different intensity represent the 2D density estimates of the tree shape statistics. Darker colors correspond to higher densities. The colors of the labels indicate which contours pertain to which analysis. The density estimates are based on n=7304 samples for RSVA and n=3002 samples for RSVB. Source data are provided as a Source Data file.

**Figure S4**: Posterior estimates of time-homogeneous predictor contributions to RSV diffusion at the continent level. The predictors include the number of passengers travelling by air between each pair of continents represented in the data set (air travel, in dark red), population size at the origin and destination location (pop size ori & pop size dest, in blue), geographic distance (geo distance, in light green), absolute differences in latitude (lat diff, in dark orange) and sample sizes at the origin and destination locations (# taxa ori & # taxa dest, in dark green). The Y-axis represents the product of the coefficient (on a log scale) and the inclusion probability for the predictors (coefficient * Inclusion). A-B: RSVA. C-D: RSVB. The plots on the left and right distinguish between analyses without and with sample size predictors respectively. The grey boxes in the violin plots represent the median and quantile estimates. The violin plots are based on n=45002 (panels A-D) post-burnin samples from the respective MCMC chains. Source data are provided as a Source Data file.

**Figure S5**: Posterior estimates of time-inhomogeneous predictor contributions to RSV global diffusion. The predictors include the number of passengers travelling by air between each pair of countries represented in the data set (air travel, in dark red), population size at the origin and destination location (pop size ori & pop size dest, in blue), geographic distance (geo distance, in light green), absolute differences in latitude (lat diff, in dark orange). The Y-axis represents the product of the coefficient (on a log scale) and the inclusion probability for the predictors (coefficient * Inclusion). A and B: RSVA estimates at the country level. C and D: RSVB estimates at the country level. E and F summarise the estimates for a single GLM-diffusion model applied to the combined RSVA and RSVB data sets at the country level. The plots on the left and right distinguish between estimates before and after 5 years prior to the most recent sampling time and are derived from a single epoch GLM-diffusion model. The grey boxes in the violin plots represent the median and quantile estimates. The violin plots are based on n=482 (panels A-B), n=45002 (panels C-D) and n=452 (panels E-F) post-burnin samples from the respective MCMC chains. Source data are provided as a Source Data file.

**Figure S6**: Unique clustering of South African lineages at the start of the 2019-2020 season (black ellipse).

**Figure S7**: Midpoint rooted ML tree for RSV A. The highly supported branch that is basal to the well supported clade containing all INFORM sequences is highlighted in blue and indicated by a green arrow. The SH-aLRT support is indicated next to the branch.

**Figure S8**: Midpoint rooted ML tree for RSV B. The highly supported branch that is basal to the well supported clade containing all INFORM sequences is highlighted in blue and indicated by a green arrow. The SH-aLRT support is indicated next to the branch.
